# CEACAM6 promotes cisplatin resistance in lung adenocarcinoma and is regulated by microRNA‐146a and microRNA‐26a

**DOI:** 10.1111/1759-7714.13558

**Published:** 2020-07-10

**Authors:** He Du, Yang Li, Rongrong Sun, Yuan Yuan, Sanyuan Sun, Youwei Zhang

**Affiliations:** ^1^ Department of Medical Oncology, Affiliated Shanghai Pulmonary Hospital Tongji University Shanghai China; ^2^ Department of Medical Oncology, Xuzhou Central Hospital Clinical School of Xuzhou Medical University Xuzhou China

**Keywords:** CEACAM6, EMT, microRNA, resistance, stemness

## Abstract

**Background:**

Carcinoembryonic antigen (CEA)‐related cell adhesion molecule 6 (CEACAM6) is a glycophosphoinositol‐anchored glycoprotein which mediates cell‐cell interactions. Here, we aimed to explore the specific functions and regulatory mechanisms of CEACAM6 on cisplatin (DDP) in lung adenocarcinoma (LUAD).

**Methods:**

RNA sequencing was performed in the DDP‐resistant A549/DDP cell line and parental A549 cell line; miRNA expression profiling of the two cell lines was analyzed using GEO data (GSE43249). Gain‐ and loss‐of‐function experiments were used to investigate the biological function of CEACAM6 in vitro. The expression status and prognostic value of CEACAM6 in LUAD were verified using The Cancer Genome Atlas (TCGA) database.

**Results:**

CEACAM6 was first screened to be one of the most significantly upregulated genes in the DDP‐resistant A549/DDP cell line compared to the parental A549 cell line. Combined with computational prediction of candidate miRNAs that target CEACAM6, miR‐146a and miR‐26a were selected and verified by qPCR and luciferase reporter assay. The knockdown of CEACAM6 expression in A549/DDP cells inhibited cell proliferation, invasion and migration, decreased the IC_50_ values of DDP, and caused a significant downregulation of N‐cadherin, vimentin, Sox2, Oct4 and GTP‐RhoA and upregulation of E‐cadherin; while CEACAM6 overexpression in A549 cells resulted in the opposite effects. Of note, both miR‐146a and miR‐26a could counteract the biological effects of CEACAM6. Furthermore, CEACAM6 mRNA expression was significantly unregulated in DDP‐resistant LUAD tissues of TCGA database.

**Conclusions:**

CEACAM6 promotes DDP resistance in LUAD by affecting the epithelial‐mesenchymal transition (EMT) phenotype and stemness, which is post‐transcriptionally regulated by miR‐146a and miR‐26a.

## Graphical abstract

• CEACAM6 expression is upregulated in the DDP‐resistant LUAD cells and tissues.

• CEACAM6 promotes DDP‐resistance via regulating EMT, stemness and RhoA activation.

• miR‐146a and miR‐26a could counteract biological effects of CEACAM6.

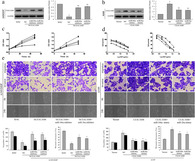

## Introduction

Lung cancer is the leading cause of cancer‐related deaths in many regions of China, and even worldwide. Lung adenocarcinoma (LUAD) occurs mostly in the peripheral airways of the lung and is currently the most increasing subtype. Although new drugs are currently being developed, platinum‐based chemotherapy remains the standard treatment for LUAD.^1^ Cisplatin (DDP), which disrupts the structure and function of DNA, is the most widely used platinum agent, but chemoresistance to DDP has become a key obstacle in the effective treatment of cancer patients. The molecular mechanisms of DDP resistance are not yet clearly defined; thus, further study is required for the advancement of LUAD treatment.

Carcinoembryonic antigen (CEA)‐related cell adhesion molecule 6 (CEACAM6) is a glycophosphoinositol‐anchored glycoprotein and belongs to the CEA family, which mediates homotypic and heterotypic cell‐cell interactions through integrin receptors.^2^ CEACAM6 is overexpressed in a wide variety of cancers and can even be used as a marker for isolating cancer stem cells in colorectal cancer.^3^ In non‐small cell lung cancer (NSCLC), LUAD expresses higher levels of CEACAM6 protein than other histologic subtypes.^4^ CEACAM6 has important functional roles in controlling the growth, migration, and invasion of LUAD cells in vitro and in vivo,^5^ suggesting that CEACAM6 is an attractive therapeutic target. Two recent studies have demonstrated the therapeutic potential of pHLIP‐mediated CEACAM6 gene silencing or anti‐CEACAM6 monoclonal antibody in LUAD.^6^, ^7^ However, the role of CEACAM6 in DDP resistance has not been investigated. In our previous studies, we found that CEACAM6 was significantly upregulated in the DDP‐resistant A549/DDP cell line compared to parental A549 LUAD cell line through RNA sequencing (data not shown).^8^ In the present study, we aimed to further explore the specific functions of CEACAM6 in DDP resistance in LUAD. MicroRNAs (miRNAs) are small, non‐coding RNAs ranging in length from 19 to 23 nucleotides that bind to the 3′‐untranslated regions (3'‐UTRs) of target mRNAs and suppress their expression and/or prohibit their translation, thereby regulating several biological processes.^9^ As potential biomarkers, miRNAs have been continuously and extensively studied; therefore we also investigated the post‐transcriptional regulation of CEACAM6 by candidate miRNAs in LUAD.

## Methods

### Cell lines and cell culture

The human LUAD cell line A549 was purchased from Shanghai Institutes for Biological Sciences, Chinese Academy of Cell Resource Center and was cultured at 37°C with 5% CO_2_ in RPMI 1640 medium (HyClone, Logan, UT, USA) with 10% fetal bovine serum (FBS) and 1% penicillin/streptomycin. The construction and culture of the DDP‐resistant A549/DDP cell line were in accordance with our previous studies. Primary LUAD cells were isolated from fresh tumors, and the culture conditions and identification of DDP sensitivity have been previously described.^10^ There were three groups of simultaneously cultured cells for each intervention, and each assay was performed in triplicate.

### Real‐time quantitative PCR (qPCR)

Total RNA was extracted using TRIzol reagent (Thermo Fisher Scientific, Waltham, MA). Reverse transcription reactions were performed using 2 μg of total RNA with a PrimeScript RT reagent kit (Cat No.: RR037A, Takara Biotechnology, Dalian, China). Gene expression levels were analyzed by an ABI 7300 thermocycler (Applied Biosystems, Foster City, CA) using a TB Green Premix Ex Taq II kit (Cat No. RR820A, TaKaRa) at the following conditions: one cycle at 95°C for 20 minutes, 40 cycles at 95°C for 5 seconds and 60°C for 30 seconds. A total of 10 ng sample cDNA and HPLC‐grade water add to 20 μL, which means to supplement the reaction system with water to 20 μL. GAPDH and β‐actin were used as reference genes. The specific primer sequences were designed using primer premier 5.0 (www.premierbiosoft.com). Data analysis was performed using the 2^‐ΔΔCt^ method to calculate the relative expression levels of the genes.

### Lentivirus infection

Oligonucleotides encoding short hairpin RNA (shRNA) targeting human CEACAM6 were cloned into the pLKO.1 lentiviral vector (Addgene, Cambridge, MA, USA). The open reading frame of CEACAM6 amplified by PCR was inserted into the pLVX lentivirus vector (Clontech, Mountain View, CA, USA). Virus packaging was performed in HEK293T cells after cotransfection of the lentiviral vectors constructed above with the packaging plasmid psPAX2 (Addgene) and envelope plasmid pMD2.G (Addgene) using Lipofectamine 2000 reagent (Invitrogen, Grand Island, NY, USA). Viruses were harvested 72 hours after transfection, and viral titers were determined. A549 cells and A549/DDP cells (1 × 10^5^) were infected with 1 × 10^6^ recombinant lentivirus‐transducing units in the presence of 6 μg/mL polybrene (Sigma, Shanghai, China). Virus‐containing culture medium was replaced with fresh RPMI‐1640 medium at 12 hours post infection, and then cells were selected using 0.5 mg/mL puromycin (Sigma) at 48 hours post infection.

### Cell viability and proliferation analysis

Cell counting kit‐8 (CCK‐8) assay was used to assess cell viability. Briefly, cells were seeded into 96‐well plates for 0–48 hours at an initial density of 2 × 10^3^ cells/well. Next, 90 μL of fresh serum‐free medium and 10 μL of CCK‐8 reagent (Beyotime, Shanghai, China) were added to each well after decanting the old medium and the plates were incubated at 37°C for 1 hour. The optical density (OD) was measured at a wavelength of 450 nm by scanning with a microplate reader (Promega). Using GraphPad Prism 5.0 (GraphPad Software, La Jolla, CA, USA), IC_50_ values were calculated by a DDP concentration response curve (concentration gradient: 0, 2, 5, 10, 20 and 40 μg/mL for 48 hours treatment period).

### Western blotting

Proteins were extracted using a Nuclear and Cytoplasmic Protein Extraction Kit (Beyotime). After centrifugation, the supernatants were collected and BCA reagent was used to determine the protein concentration. Cell protein lysates (25 μg) were separated by 10% sodium salt (SDS)‐polyacrylamide gel electrophoresis (PAGE) gels and then electroblotted from SDS‐PAGE onto polyvinylidene fluoride (PVDF) membranes (Roche Diagnostics, Mannheim, Germany). After blocking the membranes with 5% nonfat milk powder and 0.1% Tween 20 in PBS for 1 hour, the membranes were incubated with primary antibodies specific to CEACAM6, E‐cadherin (E‐cad), N‐cadherin (N‐cad), vimentin, Sox2, Oct4 and RhoA and GAPDH, all sourced from Abcam (Cambridge, UK; all used at 1:1000 dilution). After extensive washing with blocking solution, blots were exposed to horseradish peroxidase‐conjugated goat anti‐rabbit IgG. Finally, the protein bands were imaged using an enhanced chemiluminescent (ECL) substrate (Merck Millipore, Hong Kong, China).

### Luciferase reporter assay

Human embryonic kidney HEK293T cells were seeded in 96‐well plates at 1 × 10^4^ cells per well. When the cells reached 60% confluence, they were cotransfected with the wild‐type pGL3‐CEACAM6 3'‐UTR or mutated pGL3‐CEACAM6 3'‐UTR plasmid and either scrambled miRNA or miRNA mimics using Lipofectamine 2000 reagent (Invitrogen). Luciferase activity was measured with the Dual‐Luciferase Reporter Assay System (Promega) after 48 hours and expressed as the ratio between firefly and Renilla luciferase activity (Fluc/Rluc).

### Transwell assay

Cells (3 × 10^3^ cells/well) were grown in a 6‐well plate and maintained at 37°C. After 24 hours incubation, cells were serum‐starved in basic medium for 24 hours. Then, 300 μL of cell suspension adjusted to 6 × 10^4^ cells was added to the upper transwell chamber (8.0 mm with Size 24 Cluster Platel; Costar, USA) with or without 30 mL of 1 mg/mL Matrigel (BD Bioscience, San Jose, CA, USA), and 700 μL of complete medium containing 10% FBS was added to the lower chamber. The cells were then cultured for 24 hours (migration assay) or 48 hours (invasion assay) at 37°C, after which the cells were fixed with 1 mL 4% methanol for 10 minutes and stained with 1 mL 0.5% crystal violet for 30 minutes. Cells that migrated to the lower chamber and attached to the membrane were counted under a microscope (Shanghai Caikon Optical Instrument Factory, Shanghai, China).

### Wound healing assay

Cells were seeded in 35 mm tissue culture dishes (Thermo Fisher Scientific) at a density of 8 × 10^5^/dish and seeded further until they reached 100% confluence. Confluent cultures were then scratched using a pipette tip. After scratching, the well was gently washed twice with medium to remove the detached cells. Scratched cultures were imaged under a microscope at 0 and 24 hours. Cell migration was measured by the width of the scratched area at each time point in the scratched area.

### Gene set enrichment analysis (GSEA)

GSEA was performed with the JAVA program (http://software.broadinstitute.org/gsea/index.jsp) using MSigDB C2 CP: Canonical pathways gene set collection. In this study, first, based on the correlation of genes with CEACAM6 expression, GSEA generated an ordered list of all genes, and then a predefined gene set (signature of gene expression upon perturbation of certain cancer‐related genes) received an enrichment score (ES), which is a measure of statistical evidence rejecting the null hypothesis that its members are randomly distributed in the ordered list. The parameters used for the analysis were as follows. We used the “c2.all.v5.0.symbols.gmt” gene sets to run GSEA and 1000 permutations were used to calculate the *P*‐value and the permutation type was set to gene set. The maximum gene set size was fixed at 1500 genes, while the minimum size was fixed at 15 genes. The CEACAM6 expression level was used as a phenotype label, and “Metric for ranking genes” was set to Pearson correlation. All other basic and advanced fields were set to default.

### Active RhoA pull‐down assay

The cells were lysed on ice after being rinsed twice with ice‐cold PBS. Then, the lysate protein concentrations in the lysates were determined using a BCA protein assay. According to the instructions of the Active Rho Detection Kit (Cell Signaling Technology, USA), the GST‐Rhotekin‐RBD fusion protein was used to bind the activated Rho protein. The levels of the activated form of RhoA, GTP‐RhoA, were determined by western blotting using a RhoA rabbit antibody (Proteintech, USA).

### Statistical analysis

The SPSS 16.0 software system (SPSS, Chicago, IL) was used for statistical analysis. *P* < 0.05 was considered to indicate a statistically significant difference. The data are expressed as the mean ± standard error. Differences between groups were analyzed using Student's *t*‐test for comparisons between two groups or one‐way analysis of variance when more than two groups were compared. The CEACAM6 expression status and prognostic value were verified using The Cancer Genome Atlas (TCGA) data.

## Results

### Expression status of candidate miRNAs and CEACAM6 in DDP‐resistant LUAD cells and tissues

**Figure 1:**
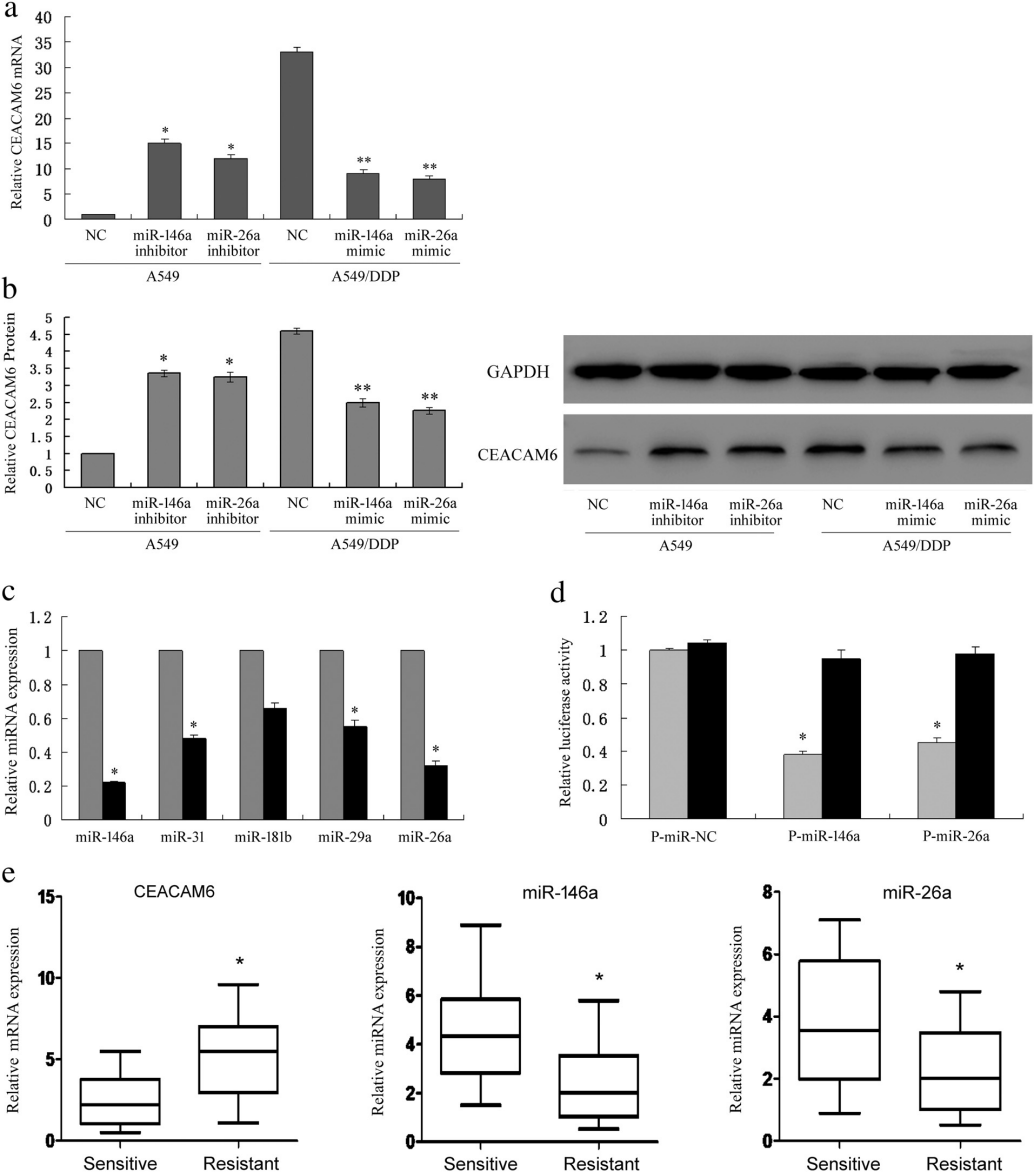
The expression status of candidate miRNAs and CEACAM6 in DDP‐resistant LUAD cells and tissues. (**a**, **b**) Expression status of CEACAM6 in A549 and A549/DDP cells at the mRNA and protein levels; *P < 0.05 vs. A549; **P < 0.05 vs. A549/DDP. (**c**) Expression levels of candidate miRNAs in A549 and A549/DDP cells (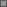) A549 (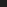) A549/DDP. (**d**) TargetScan indicates that miR‐146a and miR‐26a directly target the 3'‐UTR of CEACAM6, as verified by luciferase assays (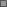) pGL3‐CEACAM6‐3'UTR‐wt (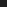) pGL3‐CEACAM6‐3'UTR‐mut. (**e**) CEACAM6, miR‐146a and miR‐26a expression in primary tumor cells; 20 LUAD samples were considered DDP‐sensitive samples (IC_50_ < 5 mg/L), and 20 samples were considered DDP‐resistant samples (IC_50_ > 10 mg/L).

We first analyzed transcriptome differences between the A549/DDP cell line and parental A549 cell line by RNA sequencing. Integrated analysis with Gene Expression Omnibus (GEO) data (GSE43493 and GSE43494) indicated that CEACAM6 is one of the most significantly upregulated members in A549/DDP cells (Table S1). The expression status of CEACAM6 in A549 and A549/DDP cells was then verified, both at the mRNA and protein levels (Fig 1a). Then, miRNA expression profiling of the A549/DDP and A549 cell lines was analyzed using GEO data (GSE43249, Table S2). Combined with computational prediction of candidate miRNAs that target CEACAM6, using a web‐based database (www.targetscan.org), miR‐146a, miR‐31, miR‐181b, miR‐26a and miR‐29a were selected for verification in our study. qPCR showed that miR‐146a and miR‐26a were the two most significantly downregulated miRNAs in A549/DDP cells compared with A549 cells (Fig 1b). Both miR‐146a and miR‐26a mimics decreased the luciferase activity of the wild‐type 3'‐UTR reporters, while that of the mutated reporters was not significantly affected, as shown by the luciferase assays (Fig 1c). Moreover, miR‐146a and miR‐26a mimics reduced the expression levels of CEACAM6 in A549/DDP cells, while miR‐146a and miR‐26a inhibitors increased CEACAM6 expression in A549 cells (Fig 1a). Using primary tumor cell culture and drug susceptibility testing, 20 LUAD samples were considered DDP‐sensitive samples (IC_50_ < 5 μg/mL), and 20 samples were considered DDP‐resistant samples (IC_50_ > 10 μg/mL). qPCR results showed that CEACAM6 expression was upregulated in DDP‐resistant tissues, while the expression levels of miR‐146a and miR‐26a were both downregulated (Fig 1d). These data indicate that miR‐146a and miR‐26a directly target the 3‐UTR of CEACAM6, thus inhibiting CEACAM6 expression and affecting DDP resistance.

### In vitro effects of CEACAM6 expression on DDP resistance

**Figure 2:**
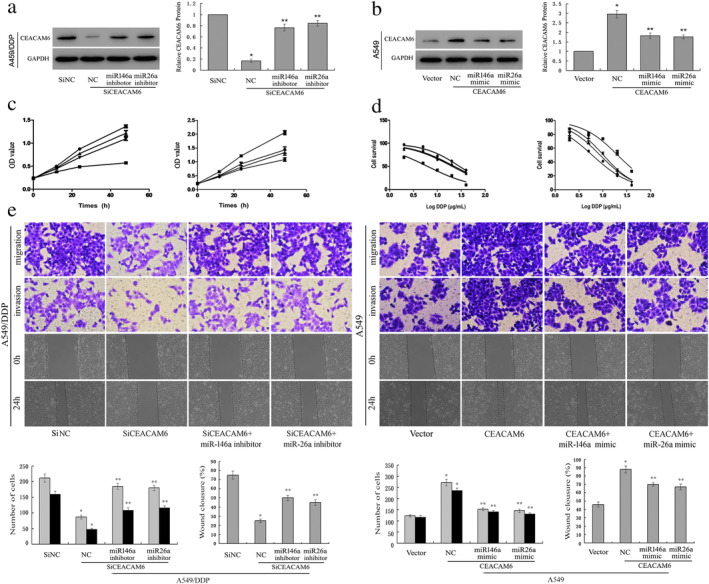
In vitro effects of CEACAM6 expression on DDP resistance. (**a**) Lentiviral vector‐meditated siRNA to knock down CEACAM6 expression in A549/DDP cells with or without miRNA inhibitors; *P < 0.05 vs. A549, **P < 0.05 vs. A549‐siCEACAM6. (**b**) CEACAM6 overexpression vector was transfected into A549 cells with or without miRNA mimics; *P < 0.05 vs. A549/DDP, **P < 0.05 vs. A549/DDP‐CEACAM6. (**c**) Cell proliferation (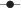) Si NC (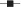) SiCEACAM6* (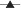) SiCEACAM6+ miR146a inhibitor ** (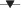) SiCEACAM6+ miR26a inhibitor ** (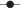) Vector (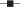) CEACAM6* (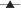) CEACAM6+ miR146a mimic ** (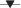) CEACAM6+ miR26a mimic ** (**d**) the IC_50_ values of DDP were analyzed using a cell counting kit‐8 (CCK‐8) assay (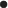) Si NC (IC50 = 29.08) (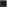) SiCEACAM6 (IC50=6.212) * (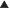) SiCEACAM6+ miR146a inhibitor (IC50 = 22.65) ** (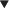) SiCEACAM6+ miR26a inhibitor (IC50 = 20.43) ** (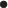) Vector (IC50 = 5.932) (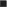) CEACAM6 (IC50 = 20.26) * (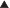) CEACAM6+ miR146a mimic (IC50 = 8.685) ** (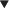) CEACAM6+ miR26a mimic (IC50 = 10.21) ** . (**e**) Cell migration and invasion were determined by transwell and wound healing assays (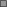) migration (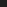) invasion (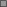) migration (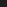) invasion.

To study the role of CEACAM6 in regulating DDP resistance, we used specific siRNAs to knockdown CEACAM6 expression in A549/DDP cells, and the CEACAM6‐overexpressing vector was transfected into A549 cells (Fig 2a). CEACAM6 knockdown in A549/DDP cells significantly inhibited cell proliferation, decreased the IC_50_ values of DDP (Fig 2b), and inhibited cell invasion and migration (Fig 2c) while CEACAM6 overexpression in A549 cells promoted cell proliferation and increased the IC_50_ values of DDP (Fig 2b), and promoted cell invasion and migration (Fig 2d). It is worth noting that both miR‐146a and miR‐26a inhibitors could reverse the in vitro effects of CEACAM6 knockdown in A549/DDP cells, while miR‐146a and miR‐26a mimics also reversed the in vitro effects of CEACAM6 overexpression in A549 cells.

### Potential mechanisms of CEACAM6 that promote DDP resistance

**Figure 3:**
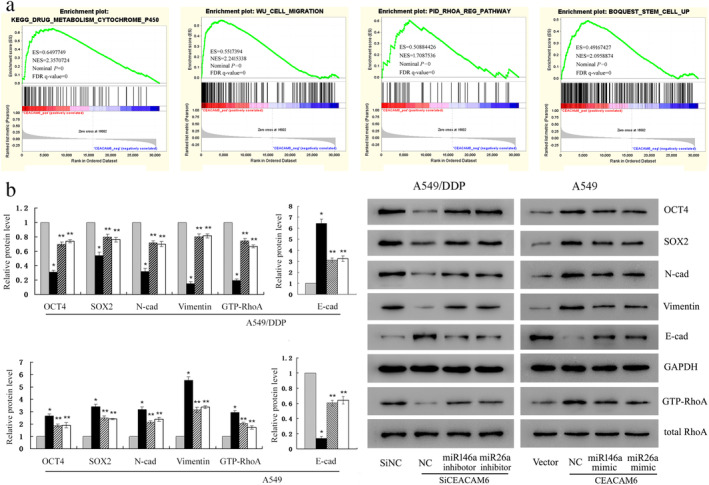
Potential mechanisms by which CEACAM6 promotes DDP resistance. (**a**) GSEA analysis showed that high expression of CEACAM6 was positively correlated with the KEGG DRUG METABOLISM CYTOCHROME P450 gene set, WU CELL MIGRATION gene set, BOQUEST STEM CELL UP, and PID RHOA REG PATHWAY gene set. (**b**) Western blot analysis of the expression levels of mesenchymal markers (N‐cadherin, vimentin), epithelial markers (E‐cadherin), stem cell transcription factors (Sox2, Oct‐4) and active RhoA (GTP‐RhoA) (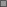) Si NC (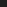) SiCEACAM6 (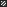) SiCEACAM6+ miR146a inhibitor (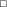) SiCEACAM6+ miR26a inhibitor (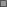) Vector (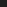) CEACAM6 (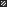) CEACAM6+ miR146a mimic (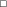) CEACAM6+ miR26a mimic; *P < 0.05 vs. A549 or A549/DDP, **P < 0.05 vs. A549‐siCEACAM6 or A549/DDP‐CEACAM6.

GSEA analysis was performed to explore the mechanisms of CEACAM6 in DDP resistance. We found that high expression of CEACAM6 was positively correlated with the KEGG_DRUG_METABOLISM_CYTOCHROME_P450 gene set (ES = 0.649 774 9, *P* = 0, FDR = 0), WU_CELL_MIGRATION gene set (ES = 0.551 739 4, *P* = 0, FDR = 0), PID_RHOA_REG_PATHWAY gene set (ES = 0.508 844 26, *P* = 0, FDR = 0), and BOQUEST_STEM_CELL_UP (ES = 0.491 674 27, *P* = 0, FDR = 0) (Fig 3a). These events are all closely related to cisplatin resistance.

To verify these processes, western blotting showed that knockdown of CEACAM6 in A549/DDP cells caused a significant downregulation of mesenchymal markers (N‐cad, vimentin), stem cell transcription factors (Sox2, Oct‐4) and active RhoA (GTP‐RhoA), and upregulation of epithelial marker (E‐cad). Conversely, CEACAM6 overexpression in A549 cells could increase the expression of N‐cad, vimentin, Sox2, Oct‐4 and GTP‐RhoA, and decrease E‐cad expression (Fig 3b). These results indicate that CEACAM6 drives epithelial‐mesenchymal transition (EMT), stemness and RhoA activation. Furthermore, both miR‐146a and miR‐26a inhibitors reversed the changes in protein expression caused by CEACAM6 knockdown in A549/DDP cells, while miR‐146a and miR‐26a mimics also counteracted the effects of CEACAM6 overexpression in A549 cells (Fig 3b).

### Expression status of CEACAM6 in LUAD

**Figure 4:**
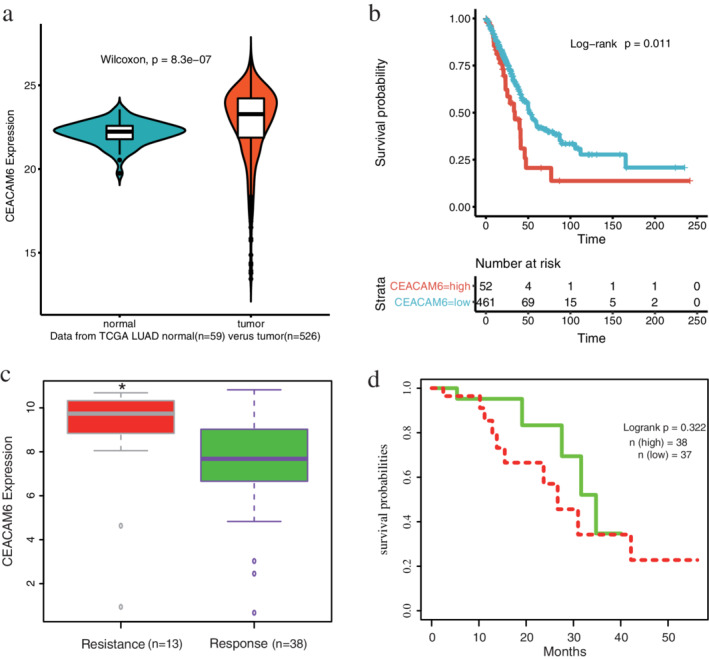
Clinical significance of CEACAM6 verified using The Cancer Genome Atlas (TCGA) data. (**a**) CEACAM6 expression was significantly upregulated in LUAD tissues (*n* = 526) compared with normal lung tissues (*n* = 59) (Wilcoxon signed‐rank test) (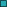) normal (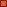) tumor. (**b**) Kaplan‐Meier survival analysis showed that high levels of CEACAM6 mRNA expression were associated with poor overall survival, strata (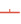) CEACAM6 = high (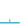) CEACAM6 = low. (**c**) There were 75 patients with LUAD who had clear DDP medication records in the TCGA database; the expression level of CEACAM6 in the DDP‐resistant group (*n* = 13) was significantly higher than that in the sensitive group (*n* = 38), Student's *t*‐test *P = 0.009. (**d**) According to the median of CEACAM6 expression, the 75 patients were divided into two groups: CEACAM6 high (*n* = 38) and CEACAM6 low (*n* = 37), their survival time was not statistically significant (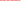) High expression (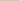) Low expression.

To better understand the clinical significance of CEACAM6 in LUAD, we further analyzed the mRNA expression of CEACAM6 using TCGA database. The results indicated that CEACAM6 expression was significantly upregulated in LUAD tissues (*n* = 526) compared with normal lung tissues (*n* = 59) (*P* < 0.001, Fig 4a), and high levels of CEACAM6 mRNA expression (52 out of the 513 patients) were associated with poor overall survival (*P* = 0.011, Fig 4b). Moreover, 75 patients had clear DDP medication records in TCGA database (Table S3). According to the response after chemotherapy, 38 patients were defined as DDP sensitive (complete remission/response), 13 patients were defined as DDP resistant (stable disease/progressive disease), and the rest is unknown or not available; the expression level of CEACAM6 in the DDP‐resistant group was significantly higher than that in the sensitive group (*P* = 0.009, Fig 4c). According to the median of CEACAM6 expression, these 75 patients were divided into two groups: CEACAM6 high (*n* = 38) and CEACAM6 low (*n* = 37), but their survival time was not statistically significant (*P* = 0.322, Fig 4d).

## Discussion

CEACAM6 is a multifunctional glycoprotein that mediates homotypic binding with other CEA family members and heterotypic binding with integrin receptors, thus activating integrin signaling and its downstream signaling cascades.^11^ Aberrant CEACAM6 expression leads to the development of several solid tumors and hematologic malignancies.^12^, ^13^, ^14^ Han *et al*.^5^ found that positive expression of CEACAM6 was identified in 86.3% (44/51) of the examined LUAD specimens, and patients with high CEACAM6 expression had significantly worse overall survival than those with low expression, according to the immunohistochemical (IHC) staining score. Functional experiments indicated that CEACAM6 enhanced the proliferation, migration, and invasion of A549 cells in vitro, and knockdown of CEACAM6 inhibited the growth of LUAD tumors in vivo. In the present study, we first found that CEACAM6 was upregulated in DDP‐resistant LUAD cells and tissues and further verified CEACAM6 promoted LUAD development and DDP resistance by accelerating proliferation, migration, and invasion. Furthermore, CEACAM6 expression is significantly upregulated in LUAD in TCGA database, and CEACAM6 mRNA overexpression is associated with poor overall survival as previously reported.^7^ Previous studies have also demonstrated that CEACAM6 overexpression in gastric cancer (GC) cells increased apoptotic resistance to 5‐FU,^15^ while gene silencing of CEACAM6 in pancreatic ductal adenocarcinoma BxPC3 cells resulted in improved sensitivity to gemcitabine through modulation of AKT activity in a Src‐dependent manner.^16^ However, how CEACAM6 is associated with drug resistance of cancer cells has not yet been fully clarified.

EMT, a process in which epithelial cells lose the apical‐basal polarity and cell–cell adhesion and transition to invasive mesenchymal cells, is involved in numerous biological and pathological processes, including embryonic development, wound healing, cancer cell metastasis and drug resistance.^17^ Cells undergoing EMT display decreased expression levels of epithelial genes (such as E‐cadherin, ZO‐1 and occludin) and increased expression levels of mesenchymal genes (such as N‐cadherin, vimentin and fibronectin). The changes in gene expression during EMT lead to numerous phenotypic changes, such as cell morphological changes, loss of adhesion and gain of stem cell‐like features. Previous studies have found that CEACAM6 induces EMT and mediates invasion and metastasis in osteosarcoma^18^ and pancreatic cancer.^19^ Additionally, CEACAM6‐overexpressing GC cells were more mesenchymal‐like and exhibited a spindle and fusiform shape, and more actin stress fibers were detected compared with the control groups, which is a process defined as EMT.^20^ As expected, the link between CEACAM6 and DDP resistance in our study is the EMT phenotype, which is represented by more migratory and invasive properties and RhoA activation. RhoA is one of members of the Rho family of small GTPase proteins and the Rho kinases are the best characterized RhoA effectors. RhoA/Rho‐kinase signaling plays a crucial role in actin cytoskeletal rearrangement and cell migration.^21^

Cancer stem cells (CSCs) are a small subpopulation of cells among the bulk tumor cells that have a high capacity for self‐renewal and differentiation and therefore play important roles in tumor growth, dissemination, metastasis and resistance to treatment.^22^ There are significant similarities between the signaling pathways that are activated during EMT and CSC formation, such as Wnt, Hedgehog and Notch signaling. Empirical evidences suggest that cells undergoing EMT have stem cell‐like properties, thus sharing key signaling pathways and drug resistance phenotypes with CSCs.^23^, ^24^ Our study indicated that CEACAM6 over‐expressing cells undergo EMT, accompanied by an elevated expression of stemness markers, which together promote DDP resistance. Of note, RhoA GTPase contributes to small intestinal stem cell maintenance by controlling YAP‐mediated EREG signaling.^25^ Yoon *et al*.^26^ also demonstrated a vital role of the RhoA pathway in diffuse gastric cancer CSCs for the maintenance of chemotherapy resistance. Thus, RhoA activation caused by CEACAM6 upregulation in our study may facilitate stemness formation of LUAD cells. However, the mechanisms by which CEACAM6 works are more complicated; for instance, its relationship with cytochrome P450 drug metabolism requires further research.

The dysregulation of miRNAs is associated with the progression and drug resistance of cancers.^27^ Several CEACAM6‐associated miRNAs have been identified. MiR‐29a/b/c specific for CEACAM6 can regulate its expression at the post‐transcriptional level in pancreatic cancer.^19^ MiR‐29a also suppresses the growth, migration, and invasion of LUAD cells by targeting CEACAM6.^5^ In the present study, we identified two new microRNAs, miR‐146a and miR‐26a, that modulate the expression of CEACAM6. Both miR‐146a and miR‐26a expression levels were downregulated in DDP‐resistant LUAD cells and tissues, and both could counteract the biological effects of CEACAM6. Since sufficient evidences have demonstrated that miR‐146a and miR‐26a increase the sensitivity of NSCLC to DDP,^28^, ^29^ these miRNAs may function by targeting CEACAM6.

In conclusion, our study demonstrates that CEACAM6 promotes DDP resistance in LUAD by affecting the EMT phenotype and stemness, which is post‐transcriptionally regulated by miR‐146a and miR‐26a, thus providing new insight into the mechanisms underlying the CEA family and contributing to developments in LUAD treatment.

## Disclosure

The authors report no conflicts of interest.

## Supporting information


**Table S1** Top 15 genes that were screened to be significantly upregulated in A549/DDP cells *vs*. A549 cells by RNA‐sequencing

**Table S2** The profiling of downregulated miRNAs in A549/DDP cells compared with A549 cells in GEO data (GSE43249)

**Table S3** Drug information and clinical response of patients with lung adenocarcinoma in TCGA database.
