# Supplementary material for: CEACAM6 promotes cisplatin resistance in lung adenocarcinoma and is regulated by microRNA‐146a and microRNA‐26a
Source: Thorac Cancer. 2020 Jul 10;11(9):2473–82. doi: 10.1111/1759-7714.13558 (PMC7471054; doi:10.1111/1759-7714.13558)
Supplement: Supplementary file 1 — Table S1 Top 15 genes that were screened to be significantly upregulated in A549/DDP cells vs. A549 cells by RNA‐sequencing [file TCA-11-2473-s001.doc]

**Table S1. Top 15 genes were screened to be significantly upregulated in A549/DDP cells vs A549 cells by RNA-sequencing**

| **seqname** | **GeneSymbol** | FC A549 vs DDP | **length** | **chrom** | **strand** | **txStart** | **txEnd** |
| --- | --- | --- | --- | --- | --- | --- | --- |
| NM_002083 | GPX2 | -33966.77533 | 1024 | chr14 | - | 65405871 | 65409531 |
| NM_005141 | FGB | -31924.07267 | 3628 | chr4 | + | 155484131 | 155492236 |
| NM_001311 | CRIP1 | -26228.9586 | 480 | chr14 | + | 105953256 | 105955122 |
| NM_001020 | RPS16 | -23155.56167 | 603 | chr19 | - | 39923848 | 39926618 |
| NM_005620 | S100A11 | -21033.013 | 595 | chr1 | - | 152004982 | 152009511 |
| NM_000146 | FTL | -20330.69833 | 889 | chr19 | + | 49468565 | 49470135 |
| NM_182715 | SYPL1 | -19099.27677 | 2149 | chr7 | - | 105730952 | 105752758 |
| NM_001899 | CST4 | -18256.195 | 736 | chr20 | - | 23666276 | 23669662 |
| NM_002276 | KRT19 | -17590.08744 | 1490 | chr17 | - | 39679869 | 39684641 |
| NM_004373 | COX6A1 | -17544.4934 | 548 | chr12 | + | 120875903 | 120878529 |
| NM_002483 | CEACAM6 | -14284.152 | 2631 | chr19 | + | 42259397 | 42276113 |
| NM_032412 | C5orf32 | -11892.70333 | 926 | chr5 | + | 139554652 | 139623372 |
| NM_001008395 | C7orf59 | -11182.098 | 613 | chr7 | + | 99746529 | 99751833 |
| NM_001312 | CRIP2 | -10936.89663 | 1236 | chr14 | + | 105941130 | 105946500 |
| NM_001145667 | GLG1 | -10324.13776 | 9339 | chr16 | - | 74481327 | 74641042 |
